# Supplementary material for: MITF regulates IDH1, NNT, and a transcriptional program protecting melanoma from reactive oxygen species
Source: Sci Rep. 2024 Sep 14;14:21527. doi: 10.1038/s41598-024-72031-9 (PMC11401838; doi:10.1038/s41598-024-72031-9)
Supplement: Supplementary file 1 — Supplementary Information. [file 41598_2024_72031_MOESM1_ESM.pdf]

Supplementary Materials for

MITF regulates IDH1, NNT, and a transcriptional program protecting melanoma from reactive oxygen species

Authors
Elisabeth Roider[1,2,3*†], Alexandra I.T. Lakatos[4,5,6, †], Alicia M. McConnell[7], Poguang Wang[8], Alina Mueller[3], Akinori Kawakami[1], Jennifer Tsoi[9,10], Botond L. Szabolcs[4,5,6], Anna A. Ascsillán[4,5,6], Yusuke Suita[1], Vivien Igras[1], Jennifer A. Lo[1], Jennifer J. Hsiao[1], Rebecca Lapides[1,12], Dorottya M.P. Pál[4,5,6], Anna S. Lengyel[4,5,6], Alexander Navarini[3], Arimichi Okazaki[2], Othon Iliopoulos[2], István Németh[13], Thomas G. Graeber[9,10,11], Leonard Zon[7], Roger W. Giese[8], Lajos V. Kemeny[1,2,4,5,6*,‡], David E. Fisher[1,2,14 *,‡]

†These authors contributed equally to this work

‡These authors contributed equally to this work

[*] Corresponding authors. Email: Elisabeth.Roider@usb.ch (E.R.), Kemeny.lajos@semmelweis.hu (L.V.K.), dfisher3@mgh.harvard.edu (D.E.F.)

This PDF file includes:

Fig. S1. MITF regulates genes involved in oxidative-reductive processes in melanoma
Fig. S2. Features of zebrafish melanoma model
Fig. S3. MALDI-TOF mass spectra
Fig. S4. Relative MITF mRNA levels after MITF silencing or overexpression

Other Supplementary Material for this manuscript includes the following:

Table S1. Supplementary Table 1. Gene sets enriched in genes downregulated after MITF KD in MALME-3M cells by DAVID. Yellow highlight indicates gene sets associated with ROS that were used to define MITF-driven redox program score in Table S3.
Table S2. Supplementary Table 2. Gene sets enriched in genes downregulated after MITF KD in MALME-3M cells by DAVID after redundant gene set removal by REVIGO.
Table S3. Supplementary Table 3. Genes and gene sets to define the MITF-driven redox program score based on the genes annotated to redox processes whose expression in MALME-3M cells changes significantly after *MITF* knockdown.

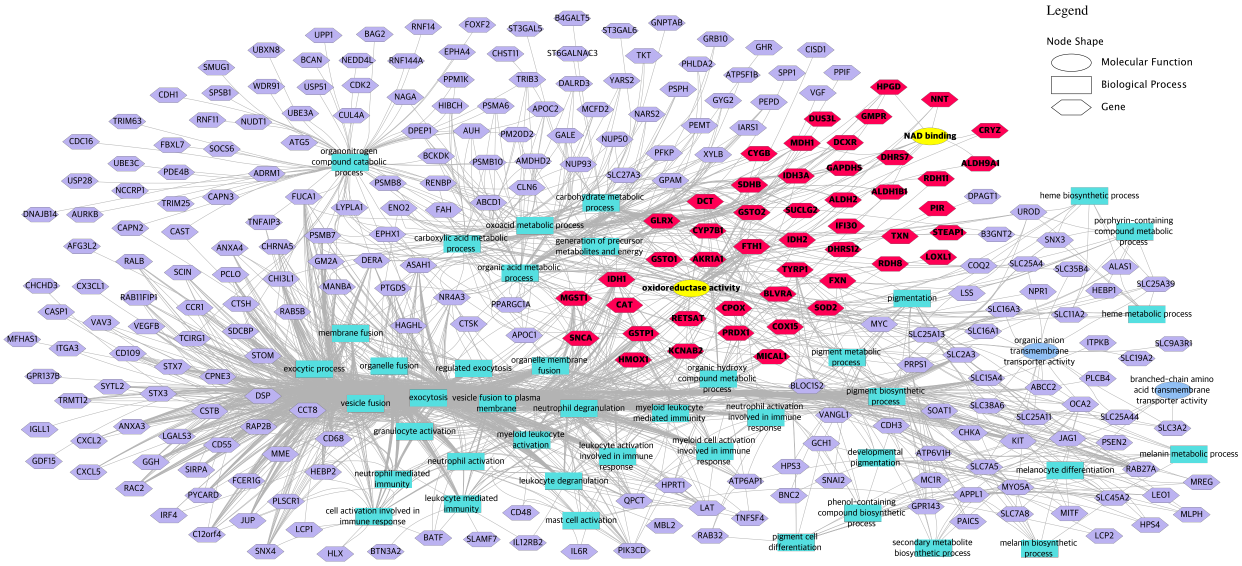

Fig. S1. **MITF regulates genes involved in oxidative-reductive processes in melanoma**. The oxidoreductase activity gene set is enriched in high MITF melanoma cell lines in short-term melanoma cultures. GSEA results were visualized by Cytoscape. Abbreviations: MITF: Microphthalmia-associated transcription factor

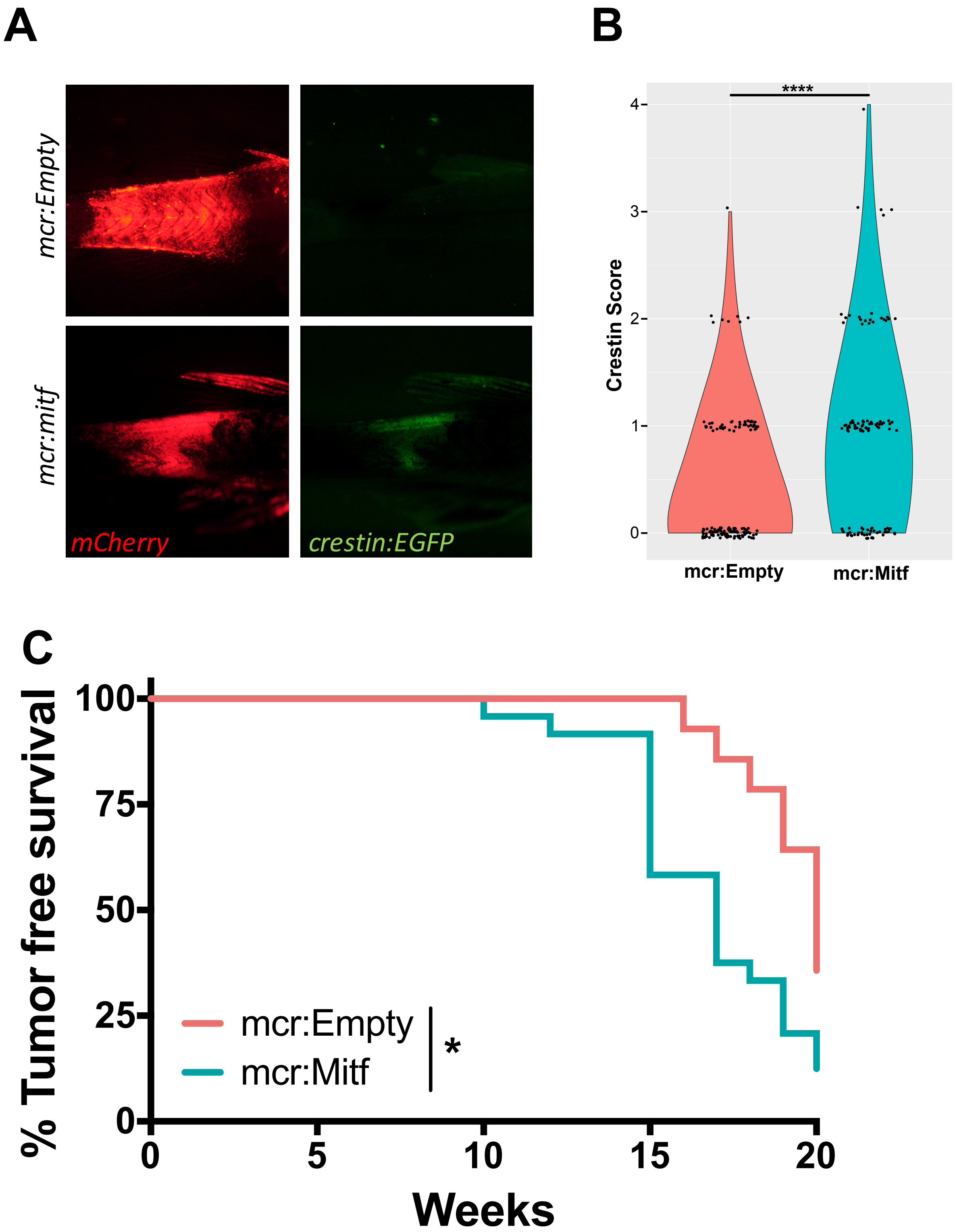

Fig. S2. **Features of zebrafish melanoma model.** Fluorescent images (**A**) and quantitation (**B**) show rescued melanocytes (*mitf:mCherry+* cells) and neural crest reactivation in early melanoma patches (*crestin:EGFP+* cells) in 6-week-old *MITF* overexpressing (*mcr:MITF*) fish compared with control

(*mcr:Empty*) fish (scored on a 0-5 scale, with 5 representing a tumor). (**C**) Kaplan-Meier curve depicting the percent of *mcr:MITF* fish with tumor-free survival compared to control *mcr:Empty* fish. The mcr:Empty vectors was used as the negative control. Abbreviations: MITF: Microphthalmia-associated transcription factor

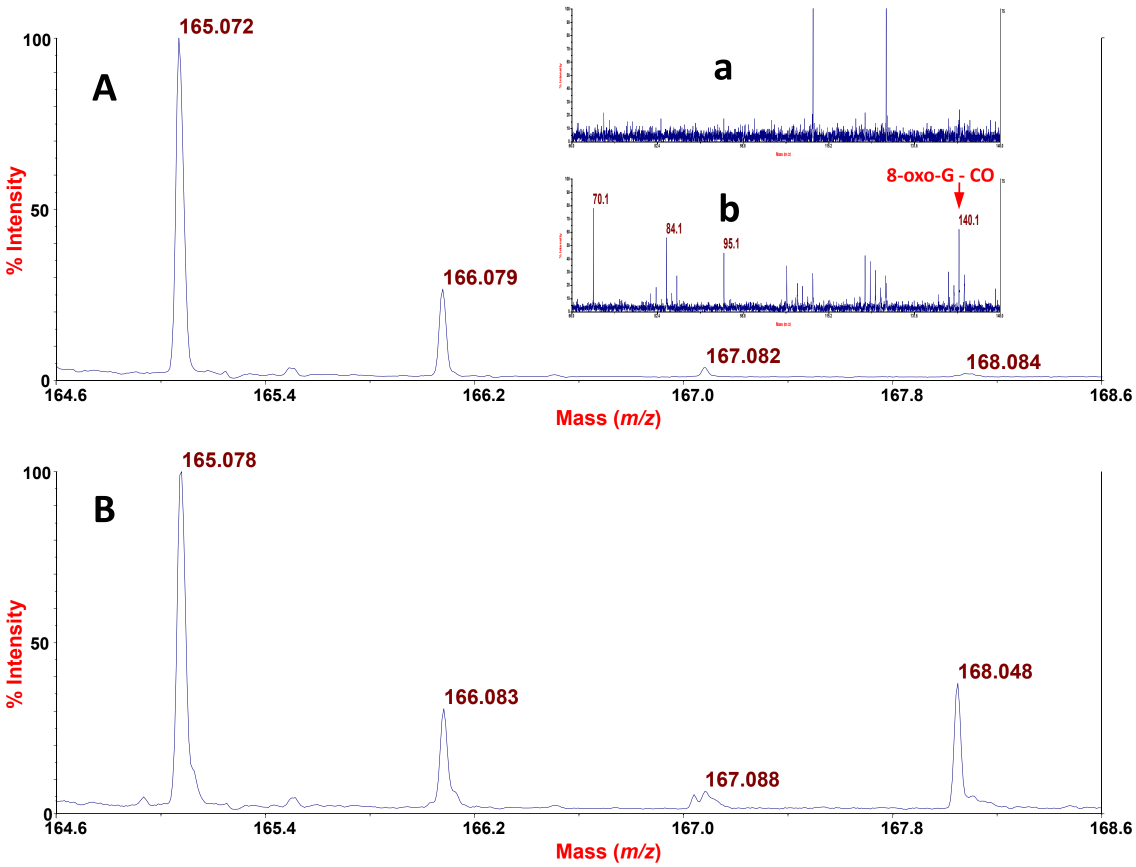

Fig. S3. **MALDI-TOF mass spectra**. (**A**) CCA matrix; (**B**) DNA from Mitf1, showing a peak for 8-oxo-G at m/z 168.048; a corresponding TOF/TOF spectrum of m/z 168 gave a peak at m/z 140.1 from loss of CO (insets a and b correspond to A and B, respectively). Abbreviations: 8- oxo-G: 8-Dihydro- 8. oxoguanine

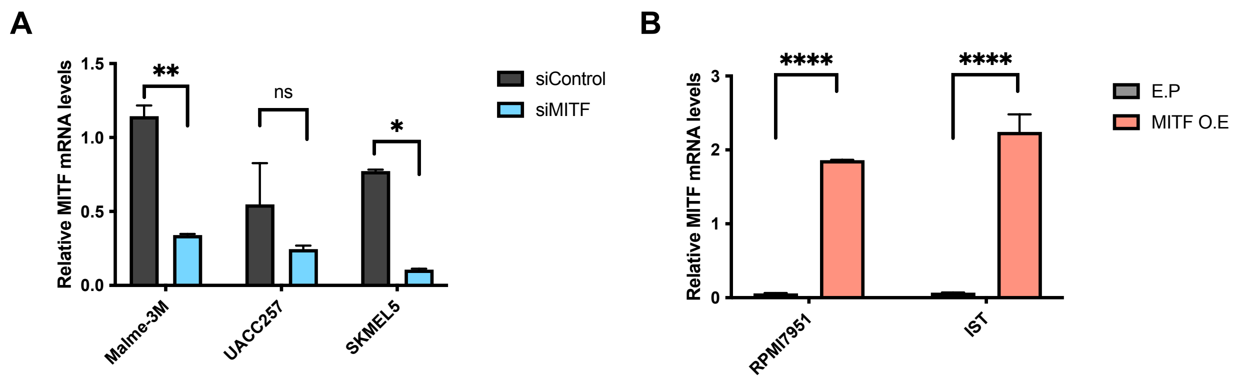

Fig. S4. **Relative MITF mRNA levels after MITF silencing or overexpression. (A)** Comparison of MITF mRNA levels following MITF gene knockdown compared to MITF control. **(B)** Comparison of MITF mRNA levels after MITF gene overexpression compared to MITF control. Non-targeting siRNA (siControl) and an empty plasmid (E.P.) were used as negative controls. All mRNA levels were normalized to RPL11. Values represent the mean ± SEM. **p< 0.05 **p < 0.01, ****p < 0.0001.* Abbreviations: MITF: Microphthalmia-associated transcription factor, E.P.: empty plasmid, O.E.: overexpression
